# Supplementary material for: ARAX: a graph-based modular reasoning tool for translational biomedicine
Source: Bioinformatics. 2023 Feb 8;39(3):btad082. doi: 10.1093/bioinformatics/btad082 (PMC10027432; doi:10.1093/bioinformatics/btad082)
Supplement: btad082_Supplementary_Data [file btad082_supplementary_data.pdf]

vignettes.md

ARAX Paper Vignettes

This page provides hyperlinks to access/view lists of biological results that are generated by running two queries through the ARAX biomedical reasoning system, each of which is described as an application "vignette" in the article *ARAX: a graph-based modular reasoning tool for translational biomedicine* by Glen et al. (2022). For more information about ARAX, or the two application vignettes (which pertain to bipolar disorder and COVID-19 disease, respectively), please see the ARAX manuscript. For more information about this page or to request an update/fix to this page, please contact Amy Glen or Stephen Ramsey.

Bipolar Disorder

ARAXi description of this query workflow:

```
add_qnode(name=DAO, key=n0)
add_qnode(categories=biolink:Polypeptide, key=n1)
add_qnode(name=bipolar disorder, key=n2)
add_qedge(subject=n0, object=n1, key=e0)
add_qedge(subject=n1, object=n2, key=e1)
expand()
overlay(action=compute_ngd, virtual_relation_label=N1, subject_qnode_key=n0,
overlay(action=compute_ngd, virtual_relation_label=N2, subject_qnode_key=n1,
resultify()
filter_results(action=limit_number_of_results, max_results=30)
```

Results

Please use your web browser to navigate to: arax.rtx.ai/?r=32764

COVID-19

ARAXi description of this query workflow:

```
add_qnode(name=REMDESIVIR, categories=biolink:SmallMolecule, key=n0)
add_qnode(name=RNA-directed RNA polymerase, categories=biolink:Protein, key=r
add_qnode(categories=[biolink:Protein, biolink:PhysiologicalProcess], key=n2)
add_qnode(name=COVID-19, categories=biolink:Disease, key=n3)
add_qedge(subject=n0, object=n1, key=e0)
add_qedge(subject=n1, object=n2, key=e1)
add_qedge(subject=n2, object=n3, key=e2)
expand(prune_threshold=500, kp_timeout=120)
overlay(action=compute_ngd, virtual_relation_label=N1, subject_qnode_key=n0,
overlay(action=compute_ngd, virtual_relation_label=N2, subject_qnode_key=n1,
overlay(action=compute_ngd, virtual_relation_label=N3, subject_qnode_key=n2,
overlay(action=fisher_exact_test, virtual_relation_label=F1, subject_qnode_ke
overlay(action=fisher_exact_test, virtual_relation_label=F2, subject_qnode_ke
resultify()
filter_results(action=limit_number_of_results, max_results=300)
```

Results

Please use your web browser to navigate to: arax.rtx.ai/?r=55721
